# The complete genome sequence and emendation of the hyperthermophilic, obligate iron-reducing archaeon *“Geoglobus ahangari”* strain 234^T^

**DOI:** 10.1186/s40793-015-0035-8

**Published:** 2015-10-09

**Authors:** Michael P. Manzella, Dawn E. Holmes, Jessica M. Rocheleau, Amanda Chung, Gemma Reguera, Kazem Kashefi

**Affiliations:** Department of Microbiology and Molecular Genetics, Michigan State University, East Lansing, MI USA; Department of Physical and Biological Sciences, Western New England University, Springfield, MA USA

**Keywords:** *Euryarchaeota*, *Archaeoglobales*, Hydrothermal vent, Guaymas basin, Fe(III) respiration, Extracellular electron transfer, Autotroph

## Abstract

*“Geoglobus ahangari”* strain 234^T^ is an obligate Fe(III)-reducing member of the *Archaeoglobales*, within the archaeal phylum *Euryarchaeota*, isolated from the Guaymas Basin hydrothermal system. It grows optimally at 88 °C by coupling the reduction of Fe(III) oxides to the oxidation of a wide range of compounds, including long-chain fatty acids, and also grows autotrophically with hydrogen and Fe(III). It is the first archaeon reported to use a direct contact mechanism for Fe(III) oxide reduction, relying on a single archaellum for locomotion, numerous curled extracellular appendages for attachment, and outer-surface heme-containing proteins for electron transfer to the insoluble Fe(III) oxides. Here we describe the annotation of the genome of *“G. ahangari”* strain 234^T^ and identify components critical to its versatility in electron donor utilization and obligate Fe(III) respiratory metabolism at high temperatures. The genome comprises a single, circular chromosome of 1,770,093 base pairs containing 2034 protein-coding genes and 52 RNA genes. In addition, emended descriptions of the genus “*Geoglobus”* and species *“G. ahangari”* are described.

**Electronic supplementary material:**

The online version of this article (doi:10.1186/s40793-015-0035-8) contains supplementary material, which is available to authorized users.

## Introduction

*“Geoglobus ahangari”* strain 234^T^ is the type strain and one of only two known members of the *Geoglobus* genus within the order *Archaeoglobales* and the family *Archaeoglobaceae*. It is an obligate Fe(III)-reducing archaeon isolated from the Guaymas Basin hydrothermal system and grows at temperatures ranging from 65–90 °C, with an optimum at about 88 °C [1]. It was the first isolate in a novel genus within the *Archaeoglobales* and the first example of a dissimilatory Fe(III)-reducer able to grow autotrophically with H_2_ [1], a metabolic trait later shown to be conserved in many hyperthermophilic Fe(III) reducers [2]. *“G. ahangari”* can also couple the reduction of soluble and insoluble Fe(III) acceptors to the oxidation of a wide range of carbon compounds including long-chain fatty acids such as stearate and palmitate, which were previously not known to be used as electron donors by archaea [1]. It was also the first hyperthermophile reported to fully oxidize acetate to CO_2_, a metabolic function once thought to occur solely in mesophilic environments [3]. Unlike the other two genera in the order *Archaeoglobales* (*Archaeoglobus* and *Ferroglobus*), which can utilize acceptors such as sulfate and nitrate [1, 4–10], the two cultured members of the genus *Geoglobus* can only use Fe(III) as an electron acceptor [1, 4]. The obligate nature of Fe(III) respiration in *Geoglobus* spp. makes the genus an attractive model to gain insights into the evolutionary mechanisms that may have led to the loss and/or gain of genes involved in the respiration of iron and other electron acceptors such as sulfur- and nitrogen-containing compounds within the *Archaeoglobales*.

*“G. ahangari”* strain 234^T^ also serves as a model organism for mechanistic studies of iron reduction at high (>85 °C) temperatures. Dissimilatory Fe(III) reduction has been extensively studied in mesophilic bacteria (reviewed in references [11, 12]). By contrast, little is known about the mechanisms that allow (hyper)thermophilic organisms to respire Fe(III) acceptors [13–18]. As previously observed in the thermophilic Gram-positive bacterium *Carboxydothermus ferrireducens* [13], *“G. ahangari”* also needs to directly contact the insoluble Fe(III) oxides to transfer respiratory electrons [14]. In *“G. ahangari”**,* cells are motile via a single archaellum, which could help in locating the oxides, and also express numerous curled extracellular appendages, which bind the mineral particles and position them close to heme-containing proteins on the outer surface of the cell to facilitate electron transfer [14]. A direct contact mechanism such as this is predicted to confer on these organisms a competitive advantage over other organisms relying on soluble mediators such as metal chelators [19] and electron shuttles [20, 21], which are energetically expensive to synthesize and are easily diluted or lost in the environment once excreted [22]. This is particularly important in hydrothermal vent systems such as the Guaymas basin chimney where *“G. ahangari”* strain 234^T^ was isolated, as vent fluids in these systems can flow through at rates as high as 2 m/s [23]. Here, we report the complete genome sequence of *“G. ahangari”* strain 234^T^ and summarize the physiological features that make this organism a good model system to study Fe(III) reduction in hot environments and to gain insights into the evolution of Fe(III) respiration in the family *Archaeoglobales*.

## Organism information

### Classification and features

**Fig. 1:**
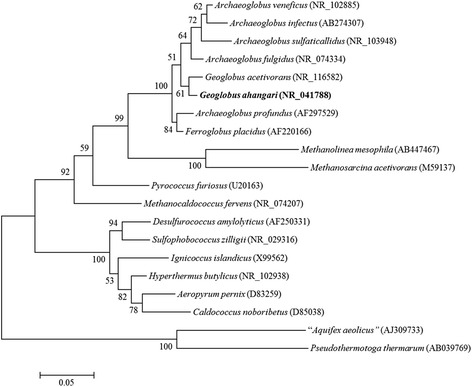
Phylogenetic tree. The phylogenetic tree was constructed with the maximum likelihood algorithm comparing the16S rRNA gene sequence from *“G. ahangari”* to other hyperthermophilic archaea. Bootstrap values were determined from 100 replicates and “*Aquifex aeolicus*” and *Pseudothermotoga thermarum* were used as outgroups

*“Geoglobus ahangari”* strain 234^T^ is a euryarchaeon originally isolated from samples obtained from a hydrothermal chimney located within the Guaymas Basin (27° N, 111° W) at a depth of 2000 m [1]. The sequence of the single 16S rRNA gene found in its genome was 99 % identical to the previously published 16S rDNA sequence (*AF220165*). The full length 16S rRNA gene (1485 bp) was used to construct a phylogenetic tree in reference to 16S rRNA gene sequences from other hyperthermophilic archaea using two thermophilic bacteria (“*Aquifex aeolicus*” and *Pseudothermotoga thermarum*) as outgroups (Fig. 1). The closest known relative was *Geoglobus acetivorans* (97 % identical), the only other known member of the *Geoglobus* genus, which also is available in pure culture [4]. Closest relatives outside the genus were other hyperthermophilic archaea within the family *Archaeoglobaceae* such as the sulfate-reducing *Archaeoglobus* species *A. fulgidus* and *A. profundus* (97 and 93 % identical, respectively) and *Ferroglobus placidus* (94 % identical), which can reduce Fe(III), thiosulfate, and nitrate [3, 5].

**Fig. 2:**
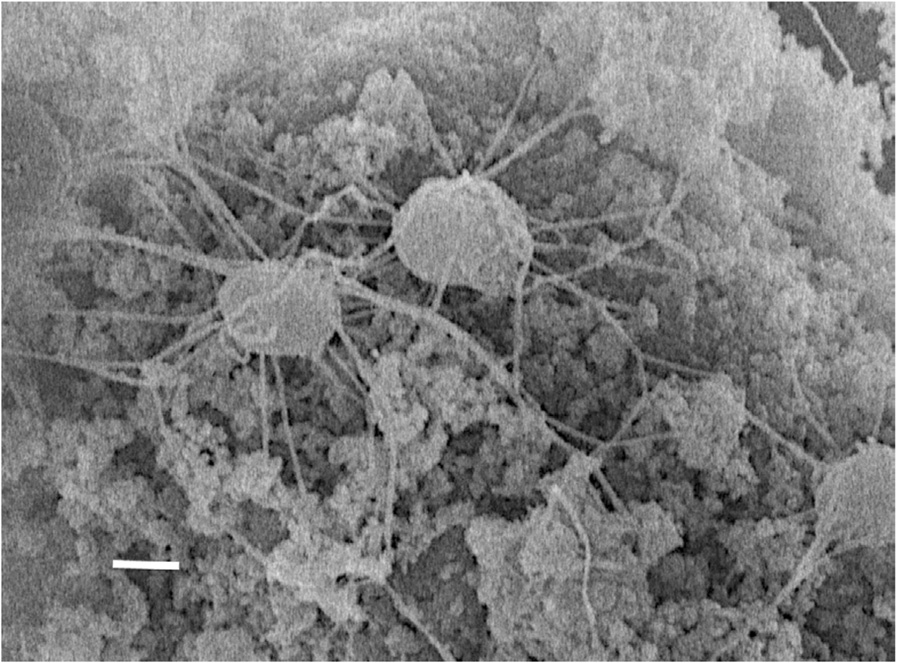
Scanning electron micrograph of cells of *“G. ahangari”* strain 234^T^ growing on insoluble Fe(III) oxides. Bar, 100 nm

Cells of *“G. ahangari”* strain 234^T^ are regular to irregular cocci, 0.3 to 0.5 μm in diameter, and usually arranged as single cells or in pairs (Fig. 2 and Table 1) [1]. Cells are motile via a single archaellum [1], but also produce abundant extracellular curled filaments when grown with both soluble and insoluble Fe(III) [14]. Though optimum growth occurs at *ca.* 88 °C, growth is observed between 65 and 90 °C [1]. Furthermore, growth was supported at pH values between 5.0 and 7.6, with an optimum at pH 7.0, and with NaCl concentrations ranging from 9 to 38 g/L, with an optimum at 19 g/L [1].

**Table 1:** Classification and general features of *“G. ahangari”* 234^T^ according to the MIGS recommendations [106]

MIGS ID	Property	Term	Evidence code^a^
	Current classification	Domain *Archaea*	TAS [107]
		Phylum *Euryarchaeota*	TAS [107, 108]
		Class *Archaeoglobi*	TAS [109]
		Order *Archaeoglobales*	TAS [110]
		Family *Archaeoglobaceae*	TAS [111]
		Genus *Geoglobus*	TAS [1]
		Species *“Geoglobus ahangari”*	TAS [1]
		Type strain 234^T^	TAS [1]
	Gram stain	Variable	NAS
	Cell shape	Irregular coccus	TAS [1]
	Motility	Motile	TAS [1]
	Sporulation	Non-sporulating	NAS
	Temperature range	65−90 °C	TAS [1]
	Optimal temperature	88 °C	TAS [1]
	pH range; Optimum	5.0−7.6 (optimum 7.0)	TAS [1]
	Carbon source	CO_2_	TAS [1]
	Energy metabolism	Chemolithoautotrophic, chemolithotrophic, chemoorganotrophic	TAS [1]
MIGS-6	Habitat	Marine geothermally heated areas	TAS [1]
MIGS-6.3	Salinity	9.0−38 g/L NaCl	TAS [1]
MIGS-22	Oxygen requirement	Anaerobe	TAS [1]
MIGS-15	Biotopic relationship	Free-living	TAS [1]
MIGS-14	Pathogenicity	Non-pathogen	NAS
	Isolation	Hydrothermal vent chimney	TAS [1]
MIGS-4	Geographic location	Guaymas Basin hydrothermal system	TAS [1]
MIGS-5	Sample collection time	Unknown	NAS
MIGS-4.1	Latitude	27° N	TAS [1]
MIGS-4.2	Longitude	111° W	TAS [1]
MIGS-4.3	Depth	2000 m	TAS [1]
MIGS-4.4	Altitude	Not applicable	

^a^Evidence codes – *IDA* Inferred from Direct Assay, *TAS* Traceable Author Statement (i.e., a direct report exists in the literature), *NAS* Non-traceable Author Statement (i.e., not directly observed for the living, isolated sample, but based on a generally accepted property for the species, or anecdotal evidence). These evidence codes are from the Gene Ontology project [112]

A distinctive feature of the metabolism of *“G. ahangari”* strain 234^T^ is its obligate nature of Fe(III) respiration, with both soluble and insoluble Fe(III) species supporting growth but the insoluble electron acceptor being preferred [1]. The obligate nature of Fe(III) reduction contrasts with the wide range of electron donors that *“G. ahangari”* can oxidize [1]. Acetate, alongside a number of other organic acids (such as propionate, butyrate, and valerate), several amino acids, and both short-chain and long-chain fatty acids were completely oxidized to CO_2_ to support growth during Fe(III) respiration [1, 3]. Furthermore, *“G. ahangari”* strain 234^T^ was also able to grow autotrophically with H_2_ as the sole electron donor and Fe(III) as the electron acceptor [1]. The main physiological features of the organism are listed in Table 1.

## Genome sequencing and annotation

### Genome project history

Based on its unique physiological characteristics [1] and use as a model system for mechanistic investigations of Fe(III) reduction by hyperthermophilic archaea [14], *“G. ahangari”* strain 234^T^ was selected for sequencing. Insights from its genome sequence and annotation provide greater understanding of the evolution of respiratory metabolisms in the *Archaeoglobales*, and, in particular, about hyperthermophilic iron reduction within the *Archaea*. The genome project information is listed in the Genomes OnLine Database (Gp0101274) [24] and the complete genome sequence has been deposited in GenBank (CP011267). A summary of the project information is shown in Table 2.

**Table 2:** Genome sequencing project information

MIGS ID	Property	Term
MIGS-31	Finishing quality	Finished
MIGS-28	Libraries used	5 independent 100 bp paired-end Illumina shotgun libraries, 150 bp paired-end Illumina shotgun library
MIGS-29	Sequencing platforms	Illumina MiSeq
MIGS-31.2	Sequencing coverage	1,977 × coverage (100 bp libraries)
		100 × (150 bp library)
MIGS-30	Assemblers	SeqMan NGen, Velvet, SeqMan Pro
MIGS-32	Gene calling method	JGI-ER, GLIMMER
	INSDC ID	CP011267
	Genbank Date of Release	May 11, 2015
	GOLD ID	Gp0101274
	NCBI project ID	258102
MIGS-13	Source material identifier	ATCC *BAA-425*, DSMZ *DSM-27542*, JCM *JCM 12378*
	Project relevance	Phylogenetic diversity, biotechnology, evolution of metal respiration in hyperthermophiles, and anaerobic degradation of hydrocarbons

### Growth conditions and genomic DNA preparation

*“G. ahangari”* strain 234^T^ was from our private culture collection and is available at the Deutsche Sammlung von Mikroorganismen und Zellkulturen (*DSM-27542*), the Japan Collection of Microorganisms (*JCM 12378*), and the American Type Culture Collection (*BAA-425*). The strain was grown in marine medium [1] with 10 mM pyruvate as the electron donor and 56 mM ferric citrate as the electron acceptor. Cultures were incubated under a N_2_:CO_2_ (80:20 %, v/v) atmosphere at 80 °C or 85 °C in the dark. Strict anaerobic techniques were used throughout the culturing and sampling experiments [25].

gDNA was extracted as previously reported for *F. placidus* [26]. Alternatively, cells were lysed with an SDS-containing lysis buffer (5 % SDS, 0.125 M EDTA, 0.5 M Tris, pH 9.4), as reported elsewhere for the preparation of whole cell extracts from *“G. ahangari”* [14], and gDNA was extracted using the MasterPure™ DNA Purification Kit (EPICENTRE® Biotechnologies), according to the manufacturer suggested guidelines.

### Genome sequencing and assembly

The finished genome of *“G. ahangari”* strain 234^T^ (CP011267) was generated from Illumina [27] draft sequences generated independently at the Research Support and Training Facility at Michigan State University, the Deep Sequencing Core Facility at the University of Massachusetts Medical School, and the Genomics Resource lab at the University of Massachusetts-Amherst. Table 2 presents the project information.

The sequencing project at the University of Massachusetts facilities used gDNA suspended in 3 ml of sonication buffer (4.95 % glycerol, 10 mM Tris–HCl (pH 8.0), 1 mM EDTA) and sonicated for 10 min (2 min on, 30 s off) using a 550 Sonic Dismembrator (Fisher Scientific). The samples were then dispensed as equal volumes into 4 tubes and mixed with 150 μl TE buffer (10 mM Tris–HCl (pH 8.0), 1 mM EDTA), 100 μl ammonium acetate (5 M), 20 μl glycogen (5 mg/ml), and 1 ml of cold (−20 °C) isopropanol. Nucleic acids were precipitated at −30 °C for 1 h, as previously described [26] and suspended in EB buffer (Qiagen) before separating the DNA fragments in the sample by agarose gel electrophoresis. DNA fragments between 300–500 bp were then purified with the QiaQuick Gel Extraction Kit (Qiagen). All steps involved in end repair, 3’ adenylation, adaptor ligation, and purification of Illumina products were performed using reagents supplied by the TruSeq DNA Sample Prep Kit (Illumina). This resulted in the construction and sequencing of five independent 100 bp paired-end Illumina shotgun libraries which generated 7,970,036, 7,970,182, 7,973,896, 7,966,671 and 3,144,785 reads totaling 3.50 Gbp. The Illumina draft sequences were assembled *de novo* with SeqMan NGen (DNASTAR) and Velvet [28] (version 1.2.10) and optimized with VelvetOptimiser (version 2.2.5). Reads were down-sampled to 500x coverage to increase the efficacy of the Velvet assembler while the complete depth of reads was used to verify the final genome assembly.

A total of 1,780,565 bp were assembled into 25 scaffolds ranging in size from 207 bp to 510,180 bp. The scaffolds were then connected by adaptor-PCR as previously described [29]. *“G. ahangari”* gDNA was subsequently digested separately by four different restriction enzymes (*Eco*RI, *Bam*HI, *Bcl*I, and *Sal*I). After a 1.5-h incubation at 37 °C (or 50 °C for *Sal*I), the restriction digests were separated by agarose gel electrophoresis and fragments between 5–10 kb were isolated and purified with the QiaQuick gel extraction kit (Qiagen). Adaptor sequences with 3′ overhangs generated by *Eco*RI, *Bam*HI, *Bcl*I, and *Sal*I at the phosphorylated 5′ ends were then ligated with T4 DNA ligase to the fragments purified from the restriction digests of *“G. ahangari”* gDNA. The adaptor sequences used were: *Eco*RI adaptor AATTCCCTATAGTGAGTCGTATTAAC** (phosphorylated at 5′ end); *Bcl*I and *Bam*HI adaptor GATCCCCTATAGTGAGTCGTATTAAC**; and finally the *Sal*I adaptor TCGACCCTATAGTGAGTCGTATTAAC**. Further assembly was performed with SeqMan Pro (DNASTAR) and primers were designed targeting the 3′ and 5′ ends of the 25 scaffolds. The adaptor ligations were diluted 100-fold and 1 μl of the diluted sample was used in PCR reactions with AccuTaq™ LA DNA Polymerase (50 μl total volume) according to manufacturer specifications (Sigma-Aldrich). Fifty reactions were performed with *“G. ahangari”*-specific primers designed from the various Illumina scaffolds and a non-phosphorylated primer that complemented the adaptor sequence on the gDNA (GTTAATACGACTCACTATAGGG). All PCR products were purified with the Qiagen PCR purification kit and sent for sequencing at the University of Massachusetts (Amherst) sequencing facility. This process was repeated until a single contig was obtained using SeqMan Pro assembly software.

The assembly was then verified against a second, independent genome assembly generated at Michigan State University. Extracted gDNA was used to construct a single Illumina shotgun library using the Illumina DNAseq Library Kit, which was sequenced in two 150 bp paired-end runs with an Illumina MiSeq at the Research Support and Training Facility at Michigan State University. The sequencing project generated 1,233,811 and 796,056 reads totaling 304.5 Mbp. Reads were quality trimmed using a combination of fastq-mcf [30] (ea-utils.1.1.2-537, using default parameters) and ConDeTri [31] (v2.2, using default parameters with the exception of hq = 33 and sc = 33) to remove low-quality reads and over-represented sequences. High-quality paired and unpaired reads were assembled using Velvet [28] (v1.2.08 using default parameters and a kmer value of 63) to generate a new assembly (1.77 Mbp, 51 contigs ≥ 1000 bp, and an N75 of 37,520 bp). The second assembly was then compared to the primary assembly to identify errors and low-coverage regions, which were subsequently resolved by PCR-amplifying and sequencing the regions of interest.

### Genome annotation

Initial genome annotation was performed by the RAST server [32], the IGS Annotation Engine [33] at the University Of Maryland School Of Medicine, and the IMG-ER platform [34]. The annotations were compared to manual annotations performed using GLIMMER [35] for gene calls and DELTA-BLAST analysis to identify conserved domains and homology to known proteins. EC numbers and COG categories were determined with a combination of DELTA-BLAST analysis of each annotated gene and the IMG-ER platform. Pseudogenes were identified using the GenePRIMP pipeline [36]. The data were used to create a consensus annotation before the final assembled genome was uploaded onto the IMG-ER platform. IMG-ER annotations were manually curated by comparison to the consensus annotation before submitting the final genome annotation.

Potential *c*-type cytochromes were selected based on the presence of *c*-type heme binding motifs (CXXCH) within the amino acid sequence as previously described [37]. Predicted subcellular localization and the presence of signal peptides and/or an N-terminal membrane helix anchor [37] was investigated by PsortB [38], PRED-TAT [39], TMPred [40], and the TMHMM Server (v. 2.0) [41]. Putative *c-*type cytochromes were then examined by BLAST analysis to determine homology to known *c*-type cytochromes in the NCBI database. The molecular weight of putative *c*-type cytochromes was estimated with the ExPASy ProtParam program [42]. The weight of the signal peptide was then subtracted from the predicted weight and 685 daltons were added for each heme-binding motif to estimate the molecular weight of the mature cytochrome. The predicted molecular weight values and subcellular localization of the mature cytochromes were compared to the masses reported for mature heme-containing proteins present in whole cells and outer-surface protein preparations of *“G. ahangari”* [14].

### Genome properties

**Fig. 3:**
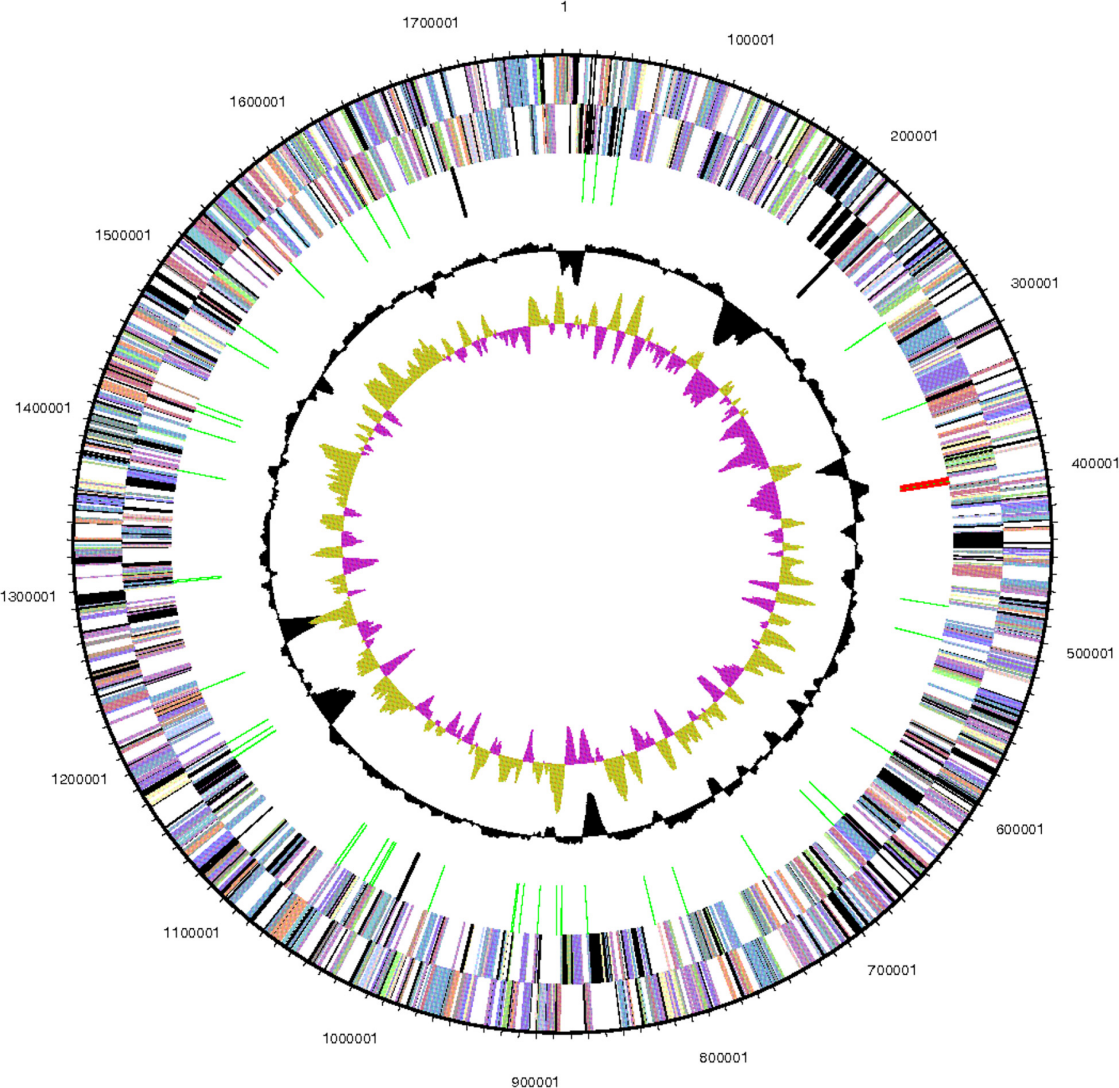
Graphical circular map of the chromosome. From outside to the center: Genes on forward strand (colored by COG categories), genes on reverse strand (colored by COG categories), RNA genes (tRNAs green, rRNAs red, other RNAs black), GC content, and GC skew

The genome of *“G. ahangari”* strain 234^T^ comprises one circular chromosome with a total size of 1,770,093 bp and does not contain any plasmids. The genome size is within the range of those reported for other members of the *Archaeoglobales* [15, 26, 43–45], and NC_015320.1. The mol percent G + C is 53.1 %, which is lower than the 58.7 % estimated experimentally via HPLC [1]. Out of the total 2072 genes annotated in the genome, 52 were identified as RNA genes and 2020 as protein-coding genes (Table 3). There are 47 pseudogenes, comprising 2.3 % of the protein-coding genes. Furthermore, 76.5 % of the predicted genes (1557) are represented by COG functional categories. Distribution of these genes and their percentage representation are listed in Fig. 3 and Table 4.

**Table 3:** Nucleotide content and gene count levels of the genome

Attribute	Value	% of total^a^
Size (bp)	1,770,093	100.0 %
Coding region (bp)	1,662,832	93.9 %
G + C content (bp)	940,071	53.1 %
Number of replicons	1	
Extrachromosomal elements	0	
Total genes	2072	100.0 %
RNA genes	52	2.5 %
rRNA operons	2	
Protein-coding genes	2034	100.0 %
Pseudogenes	47	2.3 %
Genes with function prediction	1677	82.4 %
Genes in paralog clusters	1406	69.1 %
Genes assigned to COGs	1470	72.2 %
Genes assigned Pfam domains	1667	82.0 %
Genes with signal peptides	55	2.7 %
Genes with transmembrane helices	409	20.1 %
CRISPR repeats	7	

^a^The total is based on either the size of the genome in base pairs or the total number of protein coding genes in the annotated genome

**Table 4:** Number of genes associated with the 25 general COG functional categories

Code	Value	% age^a^	Description
J	155	7.6 %	Translation, ribosomal structure and biogenesis
A	2	0.1 %	RNA processing and modification
K	68	3.3 %	Transcription
L	58	2.9 %	Replication, recombination and repair
B	6	0.3 %	Chromatin structure and dynamics
D	20	1.0 %	Cell cycle control, Cell division, chromosome partitioning
Y	0	0.0 %	Nuclear structure
V	7	0.3 %	Defense mechanisms
T	24	1.2 %	Signal transduction mechanisms
M	35	1.7 %	Cell wall/membrane biogenesis
N	15	0.7 %	Cell motility
Z	0	0.0 %	Cytoskeleton
W	0	0.0 %	Extracellular structures
U	23	1.1 %	Intracellular trafficking and secretion
O	57	2.8 %	Posttranslational modification, protein turnover, chaperones
C	160	7.9 %	Energy production and conversion
G	37	1.8 %	Carbohydrate transport and metabolism
E	139	6.8 %	Amino acid transport and metabolism
F	49	2.4 %	Nucleotide transport and metabolism
H	103	5.1 %	Coenzyme transport and metabolism
I	55	2.7 %	Lipid transport and metabolism
P	86	4.2 %	Inorganic ion transport and metabolism
Q	16	0.8 %	Secondary metabolites biosynthesis, transport and catabolism
R	244	12.0 %	General function prediction only
S	198	9.7 %	Function unknown
-	477	23.5 %	Not in COGs

^a^The total is based on the total number of protein coding genes in the genome

The preferred start codon is ATG (83.8 % of the genes), followed by GTG (10.4 %) and TTG (5.7 %). This distribution is similar to the start codon representation of the other member of the *Geoglobus* genus, *G. acetivorans* (79.4 % ATG, 11.6 % GTG, and 9.0 % TTG) [15] and the closely related archaeon *F. placidus* (82.5 % ATG, 10.2 % GTG, 6.1 % TTG, and 1.3 % other) [26]. There is one copy of each of the rRNA genes but the genes are located in two different regions of the genome: the 16S rRNA (GAH_00462) and 23S rRNA (GAH_00460) genes are in the same gene cluster and separated by a span of 139 bp encoding a single tRNA whereas the 5S rRNA (GAH_02069) is located 205,273 bp away in a region with genes coding for proteins with functions unrelated to ribosome function and biogenesis.

Almost all origins of DNA replication identified in *Archaea* to date are located in close proximity to genes coding for a homologue of the eukaryotic Cdc6 and Orc1 proteins [46]. Interestingly, we identified two genes encoding Orc1/Cdc6 family replication initiation proteins (GAH_00094 and GAH_00965) in the genome of *“G. ahangari”*, thus raising the possibility that the genome contains more than one functional origin of replication. Many archaeal replication origins consist of long intergenic sequences upstream of the *cdc6* gene containing an A/T-rich duplex unwinding element flanked by several conserved repeat motifs known as ORBs [47]. A specific ORB could not be identified in the genome when compared to other archaeal origins of replication available in the DoriC database [48]. However, the 320-bp long region upstream of GAH_00965, one of the Orc1/Cdc6 family replication initiation proteins, contains a long (111 bp) non-coding intergenic region with one AT-rich stretch and 8 direct repeats (3 TCGTGG, 3 CGTGGTC, and 2 GGGGATTA), which could function as a replication origin. Furthermore, the 580-bp region directly upstream of the other Orc1/Cdc6 family replication initiation protein (GAH_00094) lacks a non-coding intergenic region and/or AT-rich span but contains 8 direct repeats (2 GGTTGAGAAG, 3 TGAGAAG, and 3 AACATCCCG) and several “G-string” elements analogous to *ori* sites reported for haloarchaeal species [49].

## Insights from the genome

### Autotrophic growth with H_2_ as electron donor

*“G. ahangari”* strain 234^T^ was the first dissimilatory Fe(III)-reducing hyperthermophile shown to grow autotrophically with H_2_ as an electron donor [1]. In its genome, we identified genes required for the two branches of the reductive acetyl-CoA/Wood-Ljungdahl pathway [50–53], which other members of the Euryarchaeota [54], including most members of the *Archaeoglobales* [8, 45, 55–58], use for carbon fixation. A bifunctional carbon monoxide dehydrogenase/acetyl-CoA synthase complex (encoded by GAH_01139-01144, and two additional copies of the beta and maturation factors encoded by GAH_00919 and GAH_00306, respectively) are present within the genome, which could initiate carbon fixation. The bifunctional nature of this enzyme also allows it to link methyl and carbonyl branches and enable acetyl-CoA biosynthesis, as reported for methanogenic archaea [59]. Complete enzymatic pathways for alternative means of carbon fixation were not identified.

The genome of *“G. ahangari”* also contains 29 genes encoding hydrogenase subunits, maturation proteins, and a cluster of genes (*hypA*, *hypB*, *hypC*, *hypD*, and *hypE*) involved in biosynthesis and assembly of Ni-Fe hydrogenases (GAH_00190-00195). Genes coding for the large, small, and *b*-type cytochrome subunits of a Ni-Fe hydrogenase I protein (GAH_00910-00912) were identified in the genome. We also found a gene cluster (GAH_00337-00347) encoding all subunits of a NADH-quinone oxidoreductase, which transfers electrons to the quinone membrane pool and may function as the primary generator of the proton-motive force [43]. Another large cluster of hydrogenase genes (GAH_02036-02044) codes for all coenzyme F_420_ hydrogenase subunits and proteins involved in recycling coenzyme F_420_, thus replenishing the cofactor for the reductive acetyl-CoA pathway [58, 60–62]. The presence of multiple hydrogenases is not unusual in iron-reducing microorganisms and allows them to diversify the paths used to transfer electrons derived from the oxidation of H_2_ to their acceptors [63].

Autotrophic growth in methanogens can also be supported using reduced coenzyme F_420_ as an electron donor to produce methane [64]. The distinctive fluorescence emission from this coenzyme has been detected in members of the *Archaeoglobales* [7–9, 55, 57] but not in the iron-respiring *F. placidus* [5] or in *“G. ahangari”* [1]. Yet, the *“G. ahangari”* genome contains genes for all coenzyme subunits of the proteins coenzyme F_420_-reducing hydrogenase (GAH_00337 and GAH_02036-02038), coenzyme F_420_-dependent N^5^,N^10^-methylene tetrahydromethanopterin reductase (GAH_01605, GAH_01835), and F_O_ synthase (CofGH) (GAH_00662, GAH_00663) [65]. Furthermore, although *“G. ahangari”* cannot produce methane when growing autotrophically [1], its genome codes for nearly all enzymes responsible for the reduction of CO_2_ to methane [51]. Similar to *A. fulgidus*, *F. placidus*, *A. sulfaticallidus*, and *G. acetivorans*, *“G. ahangari”* has genes encoding all proteins involved in the formation of 5-methyl-tetrahydromethanopterin and a gene coding for one of the 8 subunits (MtrH) of the enzyme responsible for the transfer of a methyl group to coenzyme M (GAH_01245). Yet, the genome is missing all four genes required for a functional coenzyme M reductase, the enzyme responsible for the final step of methane production by methanogenic archaea [51]. The fact that *Archaeoglobale* genomes have nearly all of the genes involved in methanogenesis and the high level of homology that exists between genes from the reductive acetyl-CoA pathway in both *Archaeoglobales* and the methanogenic archaea suggests that the *Archaeoglobales* may have evolved from a methanogenic archaeon that lost its ability to reduce CO_2_ and produce methane over time.

### Central metabolism

**Fig. 4:**
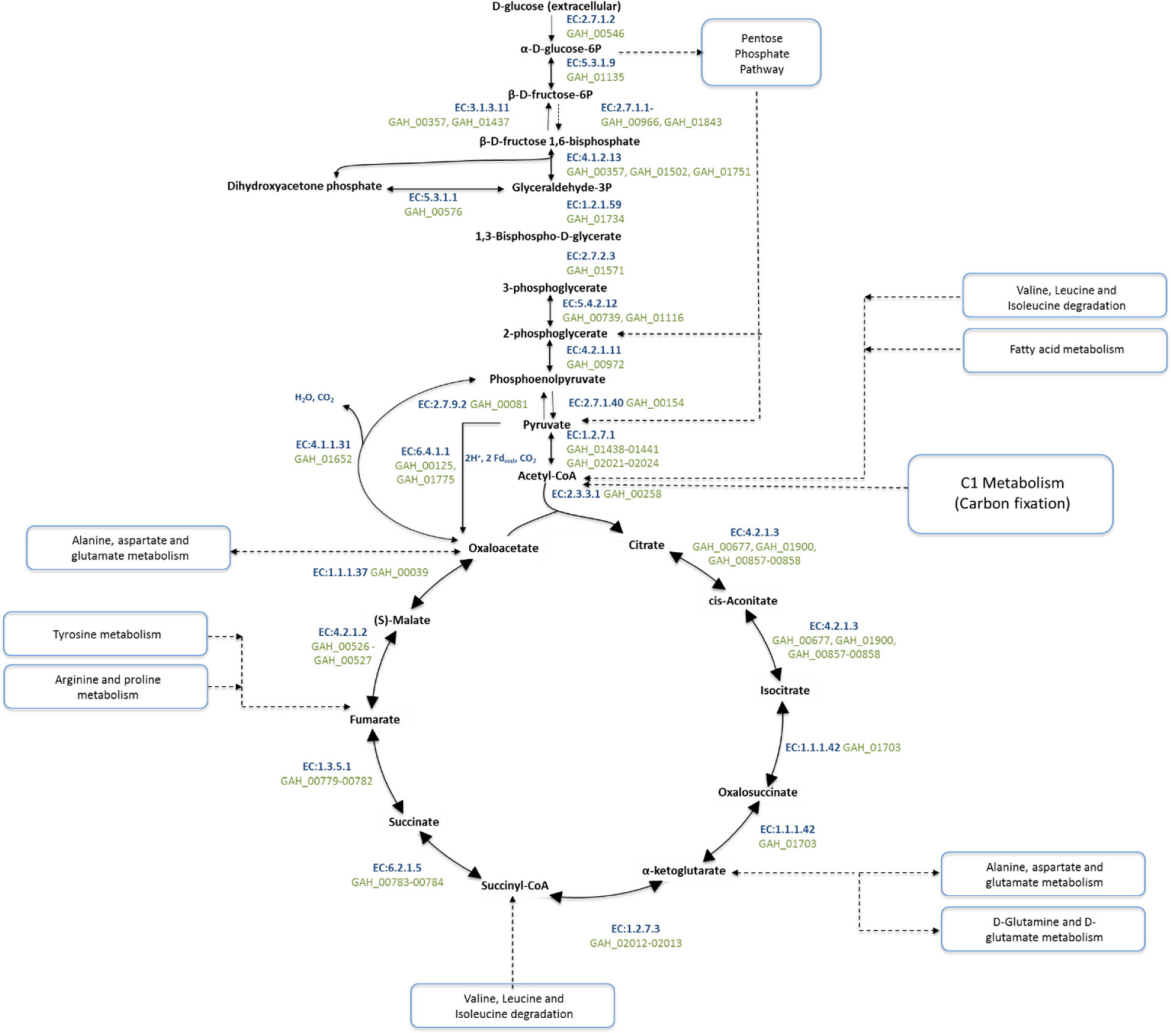
Central metabolism in *“Geoglobus ahangari”* strain 234^T^

Heterotrophic growth in *“G. ahangari”* is supported by a wide range of organic carbon compounds [1], which serve as electron donor for respiration while also providing carbon for assimilation in the central pathways. Similar to other hyperthermophilic archaeal species [66], the *“G. ahangari”* genome contains a modified Embden-Meyherhof-Parnas glycolytic pathway (Fig. 4). The initial step of glycolysis (glucose phosphorylation to glucose 6-phosphate) is carried out by an ATP-dependent archaeal hexokinase (GAH_00546) belonging to the ROK family of proteins. A gene coding for the phosphoglucose isomerase enzyme, which catalyzes the next reaction in the pathway (interconversion of the aldose in glucose 6-phosphate and the ketose in fructose 6-phosphate) was also identified (GAH_01135) and was most similar to cupin-type phosphoglucose isomerases from other anaerobic Euryarchaeota, including *Archaeoglobus fulgidus* [66].

In *A. fulgidus*, fructose 6-phosphate is phosphorylated to fructose 1,6-bisphosphate by an ADP-dependent phosphofructokinase protein (EC:2.7.1.11) [67]. However, homologs of this enzyme were not present in the genomes of *“G. ahangari”* or any other *Archaeoglobale* species sequenced to date. Instead, the genome of *“G. ahangari”* contained two genes (GAH_00966 and GAH_01843) coding for proteins with pfkB-like domains and ATP-binding sites, which are consistent with the ATP-dependent phosphofructokinases (PFK-B) of other hyperthermophilic archaea such as *Aeropyrum pernix* and *Desulfurococcus amylolyticus* [68, 69]. The genome also contains two genes (GAH_00357 and GAH_01437) encoding archaeal fructose 1,6-bisphosphatases, which catalyze the reverse reaction during gluconeogenesis but can also supply fructose 1,6-bisphosphate to the glycolytic pathway from dihydroxyacetone phosphate and *D*-glyceraldehyde 3-phosphate [70]. Furthermore, a triosephosphate isomerase (GAH_00576) was identified in the genome to catalyze the isomerization of dihydroxyacetone phosphate to D-glyceraldehyde 3-phosphate. Alternatively, GAH_01502 and GAH_01751, which encode proteins homologous to archaeal type class I fructose 1,6-bisphosphate aldolase proteins, could catalyze the conversion of fructose 1,6-bisphosphate into *D*-glyceraldehyde 3-phosphate.

The next steps in the pathway involve the oxidation of *D*-glyceraldehyde 3-phosphate and formation of 3-phosphoglycerate. The *“G. ahangari”* genome contains a homolog (GAH_00413) of a GAPOR, which in *A. fulgidus* and many other archaeal species catalyzes the irreversible oxidation of D-glyceraldehyde-3-phosphate to 3-phospho-D-glycerate bypassing the formation of the intermediate 1,3-bisphospho-*D*-glycerate [66]. In addition, the genome of *“G. ahangari”* contains genes coding for an archaeal specific type II glyceraldehyde-3-phosphate dehydrogenase (GAH_01734) and a phosphoglycerate kinase (GAH_01571), which could catalyze the formation of 3-phosphoglycerate via the 1,3-diphosphoglycerate intermediate. These two enzymes are unidirectional and involved in formation of glyceraldehyde-3-phosphate from 3-phosphoglycerate during gluconeogenesis in most hyperthermophilic archaea [66]*.*

As in *A. fulgidus* [71], *“G. ahangari”* has 2 genes coding for cofactor-independent phosphoglycerate mutase proteins (GAH_00739 and GAH_01116), which can catalyze the interconversion of 3-phospho-*D*-glycerate to 2-phospho-*D*-glycerate. Phosphoenolpyruvate is then formed by an enolase protein (GAH_00972), which is subsequently dephosphorylated to pyruvate by pyruvate kinase. Although a gene coding for the well-characterized pyruvate kinase protein is present in the close relative *F. placidus* (Ferp_0744), homologs were not identified in *“G. ahangari”* or any other *Archaeoglobale* species. Instead, the genomes of *“G. ahangari”* (GAH_00154) and all other sequenced *Archaeoglobales* species contain genes encoding PK_C superfamily proteins [15, 33, 50–52, and NC_015320.1], which have pyruvate kinase and alpha/beta domains and are homologous to an *A. fulgidus* enzyme with pyruvate kinase activity *in vitro* [72]. Pyruvate can then be converted into acetyl-CoA via pyruvate synthase (GAH_01438-01441 and GAH_02021-02024).

As in the close relative *F. placidus* [26], *“G. ahangari”* lacks genes from the oxidative pentose phosphate pathway but is predicted to circumvent this limitation [73] via the use of a complete RuMP pathway (GAH_00051 and GAH_01859). The latter results in the accumulation of formaldehyde [73], which in *“G. ahangari”* could be removed by formaldehyde-activating enzymes (GAH_00575 and GAH_00673). Ribulose 5-phosphate formed in the RuMP pathway could then be converted into ribose-5-phosphate by a ribose 5-phosphate isomerase and then into PRPP by ribose-phosphate pyrophosphokinase enzymes (GAH_00743 and GAH_00557). This supplies PRPP to various anabolic pathways such as the biosynthesis of histidine and purine/pyrimidine nucleotides.

Similar to other *Archaeoglobale* species, a complete TCA cycle is present within the *“G. ahangari”* All enzymes involved in the formation of oxaloacetate from acetyl-CoA (GAH_00258, GAH_01703, GAH_01110, GAH_02012-02013, GAH_00784-00784, GAH_00779-00782, GAH_00526-00527, and GAH_00039), including putative aconitase proteins (GAH_00857-00858) [74], were identified in the genome. Also present is a phosphoenolpyruvate carboxylase (GAH_01652), which could catalyze the reversible carboxylation of phosphoenolpyruvate to oxaloacetate, a precursor metabolite of many amino acids.

### Fatty acids as electron donors

*“G. ahangari”* strain 234^T^ was the first hyperthermophile reported to completely oxidize long-chain fatty acids anaerobically, an unsuspected capability of hyperthermophilic microorganisms prior to this discovery [1]. Long-chain fatty acids are abundant in sedimentary environments where they accumulate as byproducts of the hydrolysis of complex organic matter and the anaerobic degradation of alkanes [75, 76]. Long-chain fatty acids are also major components of crude oil [77], which is often present in environments inhabited by *Archaeoglobale* species [78]. Consistent with the ability of *Archaeoglobale* members to oxidize long-chain fatty acids, the genomes of *“G. ahangari”* and other members of the *Archaeoglobales* (*F. placidus*, *G. acetivorans*, *A. fulgidus*, and others) contain a large number of genes coding for β-oxidation pathway enzymes [15, 26, 43]. The *A. fulgidus* genome, for example, contains 57 genes encoding the 5 core proteins (discussed below) involved in β-oxidation [43]. All of these genes were used as BLAST queries against the genomes of *F. placidus* [26]*,**G. acetivorans* [15]*,* and *“G. ahangari”* and identified 39 homologous proteins in the genomes of the *F. placidus* and *G. acetivorans* and 32 in the genome of *“G. ahangari”*.

Fatty acid degradation in the *Archaeoglobales* is thought to occur in a manner similar to bacteria and mitochondria [43], with the initial step involving activation of a long chain fatty acid to a fatty acyl CoA by a fatty acyl CoA synthetase/ligase. We identified seven genes in *“G. ahangari”* coding for fatty acid CoA synthetase proteins (GAH_00420, GAH_00623, GAH_01111, GAH_01124, GAH_01769, GAH_01899, and GAH_02051). The next step in the pathway involves the oxidation of the fatty acyl-CoA to a trans-2-enoyl-CoA by acyl-CoA dehydrogenase proteins, which in *“G. ahangari”* are putatively encoded by 11 genes (GAH_00179, GAH_00421, GAH_00484, GAH_00591, GAH_01331, GAH_01442, GAH_01601, GAH_01810, and GAH_02050). A water molecule is then added to trans-2-enoyl-CoA to form (3S)-3-hydroxyacyl-CoA in a reaction catalyzed by an enoyl-CoA hydratase, which in *“G. ahangari”* could be encoded by 4 genes (GAH_00487, GAH_00802, GAH_01332, and GAH_01602). Two of these genes (GAH_00487 and GAH_01602) are in fact hybrid proteins containing an enoyl-CoA hydratase domain fused to a 3-hydroxyacyl-CoA dehydrogenase domain. Hybrid enoyl-CoA hydratase/dehydrogenase proteins such as these have been identified in other archaeal species including the *Archaeoglobales* species *G. acetivorans*, *F. placidus*, and *A. fulgidus* [15, 26, 43].

The next step in the β-oxidation pathway leads to the formation of 3-oxoacyl-CoA in an oxidation reaction that generates NADH and is catalyzed by a 3-hydroxyl-CoA dehydrogenase protein, which in *“G. ahangari”* is likely encoded by several genes (GAH_00328, GAH_00487, GAH_01600, GAH_01602 – again noting the hybrid nature of GAH_00487 and GAH_01602). Finally, acetyl-CoA is removed from the 3-oxo-acyl-CoA molecule by an acetyl-CoA acetyltransferase and is free to enter the TCA cycle. There are 8 genes in the *“G. ahangari”* genome that could catalyze this reaction (GAH_00292, GAH_00485, GAH_00625, GAH_00626, GAH_01327, GAH_01328, GAH_01886, and GAH_02049). Additional proteins involved in fatty-acid metabolism include the alpha (GAH_01318) and beta (GAH_01319) subunits of a 3-oxoacid CoA-transferase. The large number of genes dedicated to β-oxidation in *“G. ahangari”* and other species within the *Archaeoglobales* provides genomic evidence supporting the notion that long- and short-chain fatty acid oxidation is a conserved metabolic feature within the family.

### Degradation of aromatic compounds and n-alkanes

*F. placidus*, a member of the *Archaeoglobales* closely related to *“G. ahangari”*, can couple the complete oxidation of aromatic hydrocarbons to Fe(III) reduction [79–81]. The *“G. ahangari”* genome does not contain any benzoate degradation genes, further supporting the observation that it cannot utilize aromatic compounds as electron donors for growth [1]. Interestingly, *G. acetivorans*, the other member of the *Geoglobus* genus, has homologues of all genes coding for proteins of the benzoyl-CoA ligation pathway present in *F. placidus*, yet as in *“G. ahangari”* growth on aromatic hydrocarbons has not been observed in *G. acetivorans* [4, 15].

Another member of the *Archaeoglobales*, *A. fulgidus*, can also couple the oxidation of n-alkanes and n-alkenes with sulfur respiration [82, 83]. This archaeon uses an alkylsuccinate synthase and an activating protein (AssD/BssD; AF1449-1450) to oxidize saturated hydrocarbons (n-alkanes in the range of C_10_-C_21_) [83]. We identified homologs of both of these proteins in the genome of *“G. ahangari”* (GAH_01645-01646) and *G. acetivorans* (Gace_0420-0421). *A. fulgidus* can also oxidize long chain n-alk-1-enes (C_12:1_ to C_21:1_) when thiosulfate is provided as the terminal electron acceptor [82]. Although enzymes involved in the activation of alkenes by *A. fulgidus* have not been characterized, the genome of *A. fulgidus* contains a homologue of a Mo-Fe-S containing enzyme (AF0173-AF0176) [82], which in *Azoarcus**sp. EBN1* anaerobically hydroxylates a branched alkene [84]. The Mo-Fe-S enzyme consists of 4 subunits including a chaperonin-like protein, a membrane anchor heme-*b* binding subunit, an Fe-S binding subunit, and a molybdopterin-binding subunit [85]. This gene cluster was identified in the genomes *of**“G. ahangari”* (GAH_01285-01288) and *F. placidus* (Ferp_0121-0123), but not in the other member of the *Geoglobus* genus, *G. acetivorans*.

### Nitrogen compounds as electron acceptors

Except for *F. placidus* [5, 57], all of the *Archaeoglobales*, including *“G. ahangari”* [1], are unable to use nitrate or nitrite as electron acceptors for respiration [4, 6–10] (Table 5). Yet, surprisingly, the genome of *“G. ahangari”* contains several 4Fe-4S domain-containing nitrate and sulfite reductase proteins (GAH_01242 and GAH_02063) as well as all four subunits (NarGHIJ) of a nitrate reductase (GAH_01285-01288). A nitrate/nitrite transporter is also annotated in the genome (GAH_00501), though it does not cluster with genes involved in nitrate/nitrite respiration and thus may function in the transport of alternative compounds. In addition, we identified a gene in this region of the genome (GAH_01290) coding for an uncharacterized channel protein, which could potentially function as a nitrate transport protein. The presence of genes encoding both nitrate reductase proteins (NarGHIJ and NirA) combined with the inability of *“G. ahangari”* to use nitrate for respiration [1] suggests a role for these proteins in assimilatory, rather than dissimilatory, nitrate reduction [86].

**Table 5:** Terminal electron acceptors in the *Archaeoglobales*

	Electron acceptors				
Organism	Sulfate	Sulfite	Thiosulfate	Nitrate	Fe(III)
*Geoglobus spp.*	-	-	-	-	+
*Ferroglobus placidus*	-	-	+	+	+
*Archaeoglobus spp.*	+/−	+	+	-	-

Similar to *F. placidus*, the *“G. ahangari”* genome does not contain any *nir* or *nrf* genes (for the NADH- and formate-dependent nitrite reductase proteins, respectively), with the exception of several homologues of NirA (GAH_00501, GAH_00506, GAH_01242, and GAH_02063), a nitrite reductase protein that catalyzes the reduction of nitrite to ammonia and is involved in assimilatory nitrate reduction in other organisms [86]. Also missing are genes coding for nitric and nitrous oxide reductase proteins, which the genome of *F. placidus* contains [26], again supporting the observation that *“G. ahangari”* is not capable of dissimilatory nitrate reduction [1]. The lack of these enzymes helps explain the physiological separation of *“G. ahangari”* from its close phylogenetic relative *F. placidus**,* which is capable of dissimilatory nitrate reduction to N_2_O [57]. Furthermore, it is unlikely that the reduction of nitrogen-containing compounds exerts any significant selective pressure on hydrothermal vent microorganisms, as concentrations of these compounds are often low in vent systems [87].

N_2_ gas, on the other hand, is the largest reservoir of nitrogen in the ocean [87, 88] and nitrogen fixation supplies hydrothermal vent systems with nitrogen sources for assimilatory growth [87]. Ammonium is particularly abundant in the heavily sedimented Guaymas Basin hydrothermal system [89], from which *“G. ahangari”* was isolated [1], and this could select for organisms with assimilatory rather than dissimilatory nitrogen metabolisms and inhibit nitrogen fixation. Not surprisingly, the annotated genome of *“G. ahangari”* and homology searches for the primary enzymes from the nitrogen fixation pathway (*nifH*, *nifD*, and *nifK*) provided no significant hits, as previously reported for other members of the *Archaeoglobales*.

The genome does contain genes coding for a glutamine synthetase (GAH_01658), a glutamate synthase (GAH_01667-01669), and a glutamate dehydrogenase (GAH_00573 and GAH_01931). The enzymes glutamine synthetase-glutamate synthase comprise the GS-GOGAT pathway, and together with the GDH pathway, function as the two major paths for ammonium assimilation in archaea [86]. While the GDH pathway does not use ATP as an energy source, as the GS-GOGAT pathway does, it has a lower affinity for ammonium [86]. The presence of these enzymes and two ammonium transporter proteins (GAH_00438 and GAH_01767) for the formation of 2-oxoglutarate and glutamate from ammonium, is consistent with the notion that *“G. ahangari”* is under pressure to assimilate ammonium for anabolic processes.

### Sulfur compounds as electron acceptors

Most members of the *Archaeoglobales* are dissimilatory sulfate-reducing organisms and able to use several sulfur-containing compounds as electron acceptors to fuel their metabolism [5–10] (Table 5). By contrast, *“G. ahangari”* cannot couple the oxidation of electron donors that supported Fe(III) reduction to the respiration of commonly considered sulfur-containing electron acceptors such as sulfate, thiosulfate, sulfite, or S^0^ [1]. Interestingly, the genome of *“G. ahangari”* contains two genes (GAH_02067 and GAH_01481) coding for sulfate adenylyltransferase, which can initiate the first step in both the dissimilatory and assimilatory sulfate reduction pathways by catalyzing the formation of APS from ATP and inorganic sulfate. The enzyme is also present in the genome of *F. placidus* which, like *“G. ahangari”*, is unable to respire sulfate [5] (Table 5). APS can then be used as substrate in the assimilatory [90–93] or dissimilatory [94, 95] pathway, depending on the needs and capabilities of the microorganism [92]. The assimilatory pathway converts APS to the intermediate PAPS in a reaction catalyzed by an adenylsulfate kinase, which in *“G. ahangari”* is encoded by GAH_01478. The genome of *“G. ahangari”* contains genes coding for both the alpha and beta subunits of adenylsulfate reductase (GAH_02065-02066), an FAD dependent oxidoreductase protein that reduces APS to sulfite in the dissimilatory pathway. However, the genome lacks genes coding for a dissimilatory sulfite reductase (*dsrAB*), which catalyzes the reduction of sulfite to hydrogen sulfide in the final step of the dissimilatory sulfate reduction pathway [96]. Strong matches could not be found even when the alpha (AAB17213.1) and beta (AEY99618.1) subunits of the sulfite reductase from *A. fulgidus* were used as queries in manual searches.

It is interesting to note that, despite the absence of *dsrAB* genes in the genome, *“G. ahangari”* does have a nitrite and sulfite reductase 4Fe-4S domain-containing protein (GAH_02063) located in a cluster of genes involved in sulfur metabolism (GAH_02063-02067). Whether these genes code for functional proteins of the dissimilatory pathway, perhaps with electron donor/acceptor pairs not tested yet, remains to be elucidated. *F. placidus*, for example, has homologs of all of these genes, except for *dsrAB*, and it grows with thiosulfate as the sole electron acceptor when hydrogen is provided as an electron donor [5]. This capability may be due to the presence of several molybdopterin oxidoreductase proteins within the genome of *F. placidus* that show high similarity to a predicted thiosulfate reductase (NP_719592.1) from *Shewanella oneidensis*. However, strong homologs of this protein were not present in the genome of *“G. ahangari”**.*

### Fe(III) as the sole electron acceptor for respiration

The most distinctive physiological feature of *“G. ahangari”* strain 234^T^ is its dependence on Fe(III) as an electron acceptor for respiration [1]. Both insoluble Fe(III) oxides and soluble species of Fe(III), such as Fe(III) citrate, support growth, though the original isolate did not grow readily with the soluble electron acceptor and required prolonged adaptation under laboratory conditions to grow in its presence [1]. Key to the ability of *“G. ahangari”* to respire the insoluble Fe(III) oxides is the ability of the cells to locate the oxides, attach to them, and position electron carriers of the outer surface close enough to favor the transfer of electrons [14]. Hence, we examined the genome of *“G. ahangari”* for genes that code for cellular components that could be involved in motility and attachment and extracellular electron transfer.

Motility in this organism is enabled by a single flagellum [1], which in archaea is designated as an archaellum to reflect its distinct evolutionary origin [97]. Archaeal flagellar genes can be organized into one of two very well conserved clusters (*fla*1 and *fla*2) based on the type and order of genes in the cluster: *flaBC(D*/*E)FGHIJ* in *fla*1 and *flaBGFHIJ* in *fla*2 [98]. The *fla*1 clusters are exclusively found in *Euryarchaea* while *fla*2 clusters are generally associated with the *Crenarchaea*, which includes the *Desulfurococcales* and *Sulfolobales* orders, and are also present within the *Euryarchaeal* order *Archaeoglobales* [98]. Interestingly, the *Archaeoglobales* have members with both types. We identified, for example, a *fla*1 gene cluster in the genome of *“G. ahangari”* (GAH_01994-02001), as in *F. placidus* (Ferp_1456-1463) [26], while the flagellar genes of *Archaeoglobus**spp.* [15, 26, 43–45], and NC_015320.1 and *G. acetivorans* [15] were of the *fla*2 type. It has been suggested that a horizontal gene transfer (HGT) event occurred in the *Ferroglobus* lineage after divergence from the *Archaeoglobus* and *Geoglobus* lineages [15]. Yet, the presence of a *fla*1 gene cluster in the flagellated and motile *“G. ahangari”* [1]*,* when compared to the *fla*2 gene cluster found in the non-motile and non-flagellated *G. acetivorans* [4], would lend credence to a possible second HGT event within the family.

The genome of *“G. ahangari”* also encodes several glycosyltransferase genes (GAH_00218, GAH_00870, and GAH_01279) and an oligosaccharyltransferase (GAH_01455), which could glycosylate the growing archaellum [99] and post-translationally modify surface proteins, as is commonly observed in the *Archaea* [100]. However, chemotaxis proteins, which are present in nearly all sequenced members of the *Archaeoglobales* [15, 26, 43, 44], and NC_015320.1, with the exception of *A. sulfaticallidus* [45], and are typically found immediately upstream or downstream of the *fla* gene cluster, were absent in *“G. ahangari”*. The lack of chemotaxis genes in *“G. ahangari”* contrasts with their presence in most *Archaeoglobales* genomes, including *G. acetivorans* [15], the other member of the genus. Both *Geoglobus* species were isolated from hydrothermal vent chimneys: *G. acetivorans* from the Ashadze field on the Mid-Atlantic Ridge at a depth of 4100 m [4] and *“G. ahangari”* from a Guaymas Basin chimney at a depth of 2000 m [1]. The hydrothermal fluids spewed from chimneys within the Guaymas Basin system are likely enriched in nutrients after passage through the 300–400 m thick, organic-rich sediments underneath [101]. Furthermore, hydrothermal circulation at this site is high [23], which would rapidly replenish nutrients, both electron donors and fresh Fe(III) oxides, and thus organisms living in this environment may not need to utilize chemotactic mechanisms to seek out these nutrients. By contrast, hydrothermal fluids from offshore spreading systems, such as the Ashadze field, flow through thin sediment layers before reaching the chimney [101]. This likely increases the selective pressure on resident microbes to evolve chemotactic mechanisms to locate nutrients.

The genome of *“G. ahangari”* also encodes proteins potentially involved in the assembly of extracellular protein appendages such as pili. We identified, for example, a prepilin peptidase (GAH_00760), numerous type II secretion system proteins (GAH_01195-01196, GAH_00173, GAH_00290, GAH_01412-01413), and a putative twitching motility pilus retraction ATPase (GAH_00960). Homologous genes are also present in the genomes of *G. acetivorans* [15], *F. placidus* [26], and *A. fulgidus* [43]. In addition, *“G. ahangari”* has two genes encoding proteins with DUF1628 or DUF1628-like domains (GAH_01202, GAH_01671), which are associated with previously described archaeal pilin proteins [102] and present in all sequenced members of the *Archaeoglobales* [15, 26, 43–45], and NC_015320.1. Any of these proteins could be involved in the assembly of the curled extracellular appendages that *“G. ahangari”* produces to attach to Fe(III) oxides and facilitate the transfer of electrons from electron carriers located on the outer surface to the insoluble electron acceptor [14].

*“G. ahangari”* uses heme-containing proteins to transport electrons across the cell envelope and to the insoluble Fe(III) oxides [14]. The most common heme-containing proteins used by mesophilic Fe(III) reducers for extracellular electron transport are *c*-type cytochromes [103]. Archaea are known to have a variant form of the cytochrome *c* maturation (Ccm) system, whereby the CcmE protein has a CXXXY-type motif, rather than the HXXXY motif found in eukaryotic and most bacterial *c*-cytochromes, and CcmH is absent [37]. Similar to other sequenced *Archaeoglobales*, *“G. ahangari”* has an archaeal-type CcmE protein (GAH_01977), a CcmC (GAH_00620) with a tryptophan-rich motif (WG[S,T][F,Y]WNWDPRET), a CcmF protein (GAH_01976 and GAH_01093) with the motif WGGXWFWDPVEN, and a gene coding for a CcmB homolog (GAH_00449) lacking the conserved FXXDXXDGSL motif. Although previously reported archaeal cytochrome maturation pathways do not contain CcmH [37], we identified two putative CcmH proteins in the genomes of not only *“G. ahangari”* (GAH_01092 and GAH_01094), but also in *G. acetivorans* (GACE_2070 and GACE_2068) and *F. placidus* (Ferp_1362 and Ferp_1364). All of these proteins contain cysteine-rich motifs consisting of LX[S,N]C[E,D,H]C but lack the LRCXXC motif characteristic of most CcmH proteins. However, they all flank a duplicate CcmF-encoding gene found only in *“G. ahangari”* (GAH_01093), *G. acetivorans* [15], and *F. placidus* [26]*.*

In addition to having a distinct cytochrome *c* biogenesis pathway, the iron-reducing *Archaeoglobales*, *Geoglobus* and *Ferroglobus* species, also have more *c*-type cytochromes than any other archaeon, and many of these *c*-type cytochromes have multiple heme groups [15, 18, 26]. The genome of *“G. ahangari”* contains 21 genes (Table 6) encoding putative *c*-type cytochromes, 7 of which have more than 5 heme groups; *F. placidus* has 30 *c*-type cytochromes (12 with more than 5 heme groups); and *G. acetivorans* has 16 *c*-type cytochromes (8 with more than 5 heme groups). By contrast, *Archaeoglobus* species, which do not use Fe(III) electron acceptors (Table 5), have significantly fewer *c*-type cytochromes. Within this genus, the greatest number of *c*-type cytochrome encoding genes was found in the genome of *A. veneficus*, which has 16 *c*-type cytochromes (3 with more than 5 hemes). Other species such as *A. profundus* and *A. sulfaticallidus* have only 1 monoheme *c*-type cytochrome and *A. fulgidus* has 3 *c*-type cytochromes (none of which have more than 5 heme groups).

**Table 6:** Putative *c*-type cytochromes

Gene ID:	Annotation:	# of heme binding motifs:	Calculated molecular weight:	TM domains:
GAH_00015	Hypothetical protein	4	58.4	0
GAH_00283	Cytochrome c7	4	21.2^a^	1
GAH_00286	Nitrate/TMAO reductases, membrane-bound tetraheme cytochrome c subunit	12	39.1	0
GAH_00301	Putative redox-active protein (C_GCAxxG_C_C)	2	31.5	3
GAH_00504	Hypothetical protein	10	54.5	1
GAH_00505	Hypothetical protein	4	26.8	2
GAH_00506	Cytochrome c3	9	48.6	0
GAH_00507	Cytochrome c7	4	27.4^a^	1
GAH_00508	Hypothetical protein	5	28.5	1
GAH_00510	Hypothetical protein	4	27.3	1
GAH_00817	Seven times multi-haem cytochrome CxxCH	8	53.7	1
GAH_01091	Hypothetical protein	1	11.7	1
GAH_01235	Hypothetical protein	5	21.5	0
GAH_01236	Hypothetical protein	5	22.3^b^	0
GAH_01253	Hypothetical protein	4	16.9	0
GAH_01256	NapC/NirT cytochrome c family, N-terminal region	10	43.6^a^	1
GAH_01296	Cytochrome c family protein	4	17.2	1
GAH_01297	Seven times multi-haem cytochrome CxxCH	8	61.0	1
GAH_01306	Class III cytochrome C family	8	46.3	0
GAH_01534	Hypothetical protein	1	18.5	1
GAH_01700	Hypothetical protein	3	9.9	0

^a^No signal peptide detected

^b^Signal peptide detected by PRED-SIGNAL

The subcellular localization of the putative *c*-type cytochromes of *“G. ahangari”* was also investigated. The ExPASy TMPred program [42] revealed that a majority (62 %) of the *c*-type cytochromes have at least 1 transmembrane helix, consistent with their association to the cytoplasmic membrane. One of these *c*-type cytochrome proteins (GAH_00504) was predicted to be extracellular. We also identified several *c*-type cytochromes (GAH_01306, GAH_00286, GAH_01534, and GAH_01253) with predicted sizes once in mature form (46.3, 39.1, 18.5, and 16.9 kDa, respectively) matching those reported for outer-surface heme-containing proteins required for the reduction of insoluble Fe(III) oxides, but not soluble Fe(III) citrate, by *“G. ahangari”* [14] (Table 6). Hence, these 4 *c*-type cytochromes likely function as the terminal electron carriers between the cells and the oxides.

In addition to *c*-type cytochromes, we identified other potential electron carriers such as quinones, flavoproteins, and various Fe-S proteins (*i.e.* ferredoxins). We identified a number of ub iquinone/menaquinone biosynthesis proteins in the genome of *“G. ahangari”* (Additional file 1), which could create a quinone pool in the membrane to promote electron transfer. The genome also contains a great number of Fe-S binding domain proteins and ferredoxins, which could participate in electron transfer pathways (Additional file 2). Fe-S proteins and ferredoxins were also abundant in the genome of *G. acetivorans* and *F. placidus**,* which, like *“G. ahangari”**,* also utilize Fe(III) respiration as their primary metabolism. Fe-S proteins and ferredoxins are regarded as some of the most ancient of electron transfer carriers [104] and also have high thermostability [105], which is critical to ensure maximum rates of electron transfer in the hot hydrothermal vent systems. Thus, the abundance of electron carrier proteins, some known to have increased thermostability, and *c*-type cytochromes, some of them localized to the outer surface, is consistent with a mechanism evolved for efficient extracellular electron transfer in hot environments.

## Conclusions

*“G. ahangari”* strain 234^T^ is only one of three members of the *Archaeoglobales* capable of dissimilatory Fe(III) respiration. Furthermore, it is an obligate Fe(III) reducer that grows better with insoluble than soluble Fe(III) species. Consistent with this, the genome contains a large number of *c*-type cytochromes within and on the cell surface, as well as other redox-active proteins such as thermostable ferredoxin and Fe-S proteins. The paucity of *c*-type cytochromes within non-Fe(III) respiring members of the *Archaeoglobales* (*Archaeoglobus* species) is consistent with the physiological separation between these archaea and *F. placidus**,**G. acetivorans**,* and *“G. ahangari”*, which can gain energy for growth from the reduction of Fe(III) electron acceptors. Additionally, some genes required for both dissimilatory sulfate and nitrate metabolisms are absent in *“G. ahangari”* and *G. acetivorans**.* This supports the physiological separation of *Geoglobus**spp.* from *F. placidus*, which is capable of Fe(III)-, thiosulfate-, and nitrate respiration, and from *Archaeoglobus* species which are primarily sulfur-respiring organisms. Genomic data also support the reported physiological similarities between *“G. ahangari”* and other *Archaeoglobales* such as autotrophic growth with H_2_ via the reductive acetyl-CoA/Wood-Ljungdahl pathway and the use of similar electron donors, including short- and long-chain fatty acids. Noteworthy is the fact that genomic evidence supports the synthesis of the methanogenic coenzyme-F_420_ in *“G. ahangari”*, which is responsible for the characteristic fluorescence detected in all *Archaeoglobus**spp.* except for *“G. ahangari”* or *F. placidus*. Hence, the genome sequence of *“G. ahangari”* provides valuable insights into its physiology and ecology as well as into the evolution of respiration within the *Archaeoglobales*.

### Taxonomic note

The initial publication [1] of the “*Geoglobus**”* genus and “*Geoglobus ahangari**”* species was accepted for publication with extenuating circumstances at several culture-collection agencies. Thus, upon the original publication “*G. ahangari**”* strain 234^T^ was accepted only at a single agency. In addition, the G + C mol% determined from the complete genome sequence (53.1 mol%) differs from that originally published (58.7 mol%), representing a discrepancy of over 5 mol%. This publication thus warrants an emended description of the genus *Geoglobus* and the type species, “*Geoglobus ahangari**”*.

### Emended description of “*Geoglobus**”* Kashefi *et al.*

The description of the genus “*Geoglobus**”* is the one provided by Kashefi *et al.* [1], with the following modifications. In addition to the single monopolar flagellum, numerous curled filaments can be seen per cell [14]. The G + C content of the genomic DNA of the type species is 53.1 mol%.

### Emended description of “*Geoglobus ahangari**”* Kashefi *et al.*

The description of the species “*Geoglobus ahangari**”* is the one provided by Kashefi *et al.* [1, 2], with the following modifications. The type strain is strain 234^T^ and has been deposited at three culture collection agencies, which include the Deutsche Sammlung von Mikroorganismen und Zellkulturen (*DSM-27542*), the Japan Collection of Microorganisms (*JCM 12378*), and the American Type Culture Collection (*BAA-425*).

## Additional files

Additional file 1: 
**Ub iquinone and menaquinone biosynthesis proteins present in the genome of** ***G. ahangari.*** Ub iquinone and menaquinone biosynthesis proteins identified within the genome of *G. ahangari* strain 234^T^. (DOCX 16 kb) Additional file 2: 
**Fe-S binding domain proteins and ferredoxins within the genome of** ***G. ahangari.*** Fe-S binding domain proteins and ferredoxins identified within the genome of *G. ahangari* strain 234^T^. (DOCX 16 kb)

## Abbreviations

SDS: Sodium dodecyl sulfate
EDTA: Ethylenediaminetetraacetic acid
TE: Tris-EDTA
EB: Elution buffer
RAST: Rapid Annotation using Subsystem Technology
IGS: Institute for Genome Sciences
IMG-ER: Integrated Microbial Genomes – Expert Review
EC: Enzyme Commission
COG: Clusters of Orthologous Group
HPLC: High performance liquid chromatography
ORB: Origin recognition box
ROK: Repressor protein, open reading frame, sugar kinase
GAPOR: Glyceraldehyde-3-phosphate:ferredoxin oxidoreductase
RuMP: Ribulose monophosphate
PRPP: 5-phosphoribosyl diphosphate
TCA: Tricarboxylic acid
Fe-S: Iron-sulfur
GS-GOGAT: Glutamine synthetase-glutamate synthase
GDH: Glutamate dehydrogenase
APS: Adenosine 5′-phosphosulfate
PAPS: 3′-phosphoadenylyl sulfate
