# Supplementary material for: The complete genome sequence and emendation of the hyperthermophilic, obligate iron-reducing archaeon “Geoglobus ahangari” strain 234T
Source: Stand Genomic Sci. 2015 Oct 9;10:77. doi: 10.1186/s40793-015-0035-8 (PMC4600277; doi:10.1186/s40793-015-0035-8)
Supplement: Additional file 1: — Ub iquinone and menaquinone biosynthesis proteins present in the genome of G. ahangari. Ub iquinone and menaquinone biosynthesis proteins identified within the genome of G. ahangari strain 234T. (DOCX 16 kb) [file 40793_2015_35_MOESM1_ESM.docx]

**The complete genome sequence of the hyperthermophilic, obligate iron-reducing archaeon *Geoglobus ahangari* strain 234^T^**

Michael P. Manzella ^1^, Dawn E. Holmes ^2^, Jessica M. Rocheleau ^2^, Amanda Chung ^2^, Gemma Reguera ^1^, and Kazem Kashefi ^1^*

* Corresponding author: Kazem Kashefi

[kashefi@msu.edu](mailto:markus.goeker@dsmz.de)

^1^ Department of Microbiology and Molecular Genetics, Michigan State University, MI, USA

^2^ Department of Physical and Biological Sciences, Western New England University, MA, USA

**Additional file 1.** Uniquinone and menaquinone biosynthesis proteins present in the genome of *G. ahangari*

| **Protein name** | **Abbreviation** | **Homologs in *G. ahangari*** |
| --- | --- | --- |
| 1,4-dihydroxy-2-naphthoate octaprenyltransferase | menA | GAH_01919 |
| naphthoate synthase | menB | GAH_01602, GAH_00487, GAH_01332 |
| chorismate dehydratase | mqnA | GAH_00872 |
| cyclic dehypoxanthinyl futalosine synthase | mqnC | GAH_00873, GAH_00871, GAH_00663 |
| 1,4-dihydroxy-6-naphthoate synthase | mqnD | GAH_02003 |
| aminodeoxyfutalosine synthase | mqnE | GAH_00871, GAH_00873, GAH_00663 |
| 4-hydroxybenzoate polyprenyltransferase | ubiA | GAH_00304, GAH_00157 |
| phenylphosphate carboxylase subunit beta | ubiD | GAH_01625, GAH_00517 |
| 4-hydroxy-3-polyprenylbenzoate decarboxylase | ubiD | GAH_01570 |
| UbiD family decarboxylase | ubiD | GAH_01625 |
| demethylmenaquinone methyltransferase | ubiE/menG | GAH_00100, GAH_01336, GAH_00796 |
