# Supplementary material for: The complete genome sequence and emendation of the hyperthermophilic, obligate iron-reducing archaeon “Geoglobus ahangari” strain 234T
Source: Stand Genomic Sci. 2015 Oct 9;10:77. doi: 10.1186/s40793-015-0035-8 (PMC4600277; doi:10.1186/s40793-015-0035-8)
Supplement: Additional file 2: — Fe-S binding domain proteins and ferredoxins within the genome of G. ahangari. Fe-S binding domain proteins and ferredoxins identified within the genome of G. ahangari strain 234T. (DOCX 16 kb) [file 40793_2015_35_MOESM2_ESM.docx]

**The complete genome sequence of the hyperthermophilic, obligate iron-reducing archaeon *Geoglobus ahangari* strain 234^T^**

Michael P. Manzella ^1^, Dawn E. Holmes ^2^, Jessica M. Rocheleau ^2^, Amanda Chung ^2^, Gemma Reguera ^1^, and Kazem Kashefi ^1^*

* Corresponding author: Kazem Kashefi

[kashefi@msu.edu](mailto:markus.goeker@dsmz.de)

^1^ Department of Microbiology and Molecular Genetics, Michigan State University, MI, USA

^2^ Department of Physical and Biological Sciences, Western New England University, MA, USA

**Additional file 2.** Fe-S binding domain proteins and ferredoxins within the genome of *G. ahangari*

| **Locus Tag** | **Gene Product Name** |
| --- | --- |
| GAH_00040 | Ferredoxin-like domain protein |
| GAH_00110 | Uncharacterized Fe-S oxidoreductase |
| GAH_00113 | Fe-S oxidoreductase |
| GAH_00114 | Predicted Fe-S oxidoreductases |
| GAH_00137 | Fe-S oxidoreductase |
| GAH_00138 | Fe-S oxidoreductase |
| GAH_00140 | Iron-sulfur cluster-binding protein |
| GAH_00158 | Ferredoxin-like domain protein |
| GAH_00165 | Predicted Fe-S oxidoreductases |
| GAH_00413 | Aldehyde:ferredoxin oxidoreductase |
| GAH_00488 | Electron transfer flavoprotein beta |
| GAH_00537 | Heterodisulfide reductase, subunit A and polyferredoxins |
| GAH_00538 | Iron-sulfur cluster-binding oxidoreductase |
| GAH_00570 | Pyruvate ferredoxin oxidoreductase |
| GAH_00615 | Uncharacterized Fe-S protein PflX |
| GAH_00622 | Radical SAM domain iron-sulfur cluster-binding oxidoreductase with cobamide-binding-like domain |
| GAH_00678 | Predicted Fe-S-cluster oxidoreductase |
| GAH_00684 | Ferredoxin |
| GAH_00822 | Fe-S oxidoreductase |
| GAH_00845 | Ferredoxin |
| GAH_00886 | Fe-S oxidoreductase |
| GAH_00924 | Ferredoxin |
| GAH_01011 | Indolepyruvate ferredoxin oxidoreductase |
| GAH_01136 | 4Fe-4S binding domain/Putative Fe-S cluster |
| GAH_01180 | Iron-sulfur cluster-binding oxidoreductase |
| GAH_01225 | Ferredoxin |
| GAH_01254 | Fe-S-cluster-containing hydrogenase |
| GAH_01276 | Fe-S oxidoreductase |
| GAH_01286 | Fe-S-cluster-containing hydrogenase |
| GAH_01295 | Fe-S-cluster-containing hydrogenase |
| GAH_01344 | Predicted Fe-S oxidoreductase |
| GAH_01351 | Uncharacterized Fe-S center protein |
| GAH_01440 | Ferredoxin |
| GAH_01646 | Ferredoxin |
| GAH_01669 | Ferredoxin |
| GAH_01685 | Rubredoxin |
| GAH_01686 | Uncharacterized flavoproteins |
| GAH_01728 | Aldehyde:ferredoxin oxidoreductase |
| GAH_01737 | Predicted Fe-S oxidoreductases |
| GAH_01738 | Aldehyde:ferredoxin oxidoreductase |
| GAH_01822 | Aldehyde:ferredoxin oxidoreductase |
| GAH_01856 | Aldehyde:ferredoxin oxidoreductase |
| GAH_01866 | Ferredoxin-thioredoxin reductase |
| GAH_01870 | Fe-S oxidoreductase |
| GAH_01921 | Dehydrogenases (flavoproteins) |
| GAH_01948 | Ferredoxin |
| GAH_01960 | Ferredoxin |
| GAH_01981 | Fe-S oxidoreductase |
| GAH_02012 | Pyruvate ferredoxin oxidoreductase |
| GAH_02033 | Heterodisulfide reductase, subunit A |
